# Design of Polycation-Functionalized Resveratrol Nanocrystals for Intranasal Administration

**DOI:** 10.3390/pharmaceutics17101346

**Published:** 2025-10-18

**Authors:** Angela Bonaccorso, Elide Zingale, Giuseppe Caruso, Anna Privitera, Claudia Carbone, Maria Josè Lo Faro, Filippo Caraci, Teresa Musumeci, Rosario Pignatello

**Affiliations:** 1 Department of Drug and Health Sciences, University of Catania, 95125 Catania, Italy; elide.zingale@phd.unict.it (E.Z.); anna.privitera1@phd.unict.it (A.P.); ccarbone@unict.it (C.C.); fcaraci@unict.it (F.C.); teresa.musumeci@unict.it (T.M.); r.pignatello@unict.it (R.P.); 2 NANOMED-Research Centre for Nanomedicine and Pharmaceutical Nanotechnology, University of Catania, 95125 Catania, Italy; 3 CERNUT-Research Centre on Nutraceuticals and Health Products, University of Catania, 95125 Catania, Italy; 4 Departmental Faculty of Medicine, UniCamillus—Saint Camillus International University of Health and Medical Sciences, 00131 Rome, Italy; giuseppe.caruso@unicamillus.org; 5 IRCCS San Camillo Hospital, Lido di Venezia, 30126 Venezia, Italy; 6 Department of Biomedical and Biotechnological Sciences, University of Catania, 95123 Catania, Italy; 7 Dipartimento di Fisica e Astronomia “Ettore Majorana”, University of Catania, 95123 Catania, Italy; mariajose.lofaro@unict.it; 8 CNR-IMM UoS Catania, Istituto per La Microelettronica e Microsistemi, 95123 Catania, Italy; 9 Unit of Neuropharmacology and Translational Neurosciences, Oasi Research Institute-IRCCS, 94018 Troina, Italy

**Keywords:** quality by design, nanotechnology, natural compound, nose-to-brain, cell viability

## Abstract

**Background/Objectives:** Nanocrystals (NCs) are a relatively underexplored yet adaptable platform with broad potential for various applications. Currently, the surface modification of NCs leads to the development of versatile platforms capable of enhancing targeted delivery potential and supporting the advancement of precision medicine. With this in mind, this study focused on the design and surface functionalization of a resveratrol (RSV) NC selected for its antioxidant and neuroprotective effects. **Methods**: The design of the RSV NC was assessed by the Quality by Design approach. With the aim of intranasal administration, we assessed the RSV NC functionalization with a cationic poly (amino acid) belonging to the class of cell-penetrating peptides. Both naked and surface-modified RSV nanosuspensions were characterized in terms of mucoadhesion, behavior in artificial cerebrospinal fluid, crystallinity, solubility, and storage stability. The scavenging activity (%) of neat RSV and its nanosized forms was measured using the DPPH assay. **Results**: RSV NCs were successfully designed, producing truncated cubic crystals (~240 nm) with an ~80% drug content. Functionalization was efficiently achieved with poly-l-arginine hydrochloride as revealed by DSC and FTIR and resulted in a positively charged nanosuspension. Nanonization technology improved drug solubility in water and did not affect RSV scavenging activity. Technological characterization demonstrated that both nanosuspensions present suitable properties for intranasal administration in terms of particle size, mucoadhesive tendency, and stability in artificial cerebrospinal fluid. An MTT assay revealed the safety of all treatments in human microglia (HMC3) cells. **Conclusions**: RSV NCs’ functionalization enhanced their brain delivery potential, establishing a promising platform to improve therapeutic outcomes in neurodegenerative diseases.

## 1. Introduction

A growing number of novel drug candidates exhibit low aqueous solubility, classifying them as Class II or IV within the Biopharmaceutics Classification System (BCS). This inherent property significantly impedes their potential for therapeutic application, often necessitating extensive and laborious reformulation efforts. While traditional solubilization techniques such as salt formation, co-crystallization, or cyclodextrin complexation have been employed, advancements in nanotechnology have introduced innovative solutions [1]. Notably, nanocrystals (NCs) have gained attention as a promising approach to overcome formulation challenges of poorly water-soluble drugs, largely due to their straightforward composition. NCs are particles composed of a 100% pure drug, surrounded by a stabilizer and suspended in an aqueous medium [2]. Owing to their high drug loading, NCs can maintain potent therapeutic concentrations to elicit the desired pharmacological effects. The early 2000s marked the undeniable peak of commercial success for nanosizing technologies, with the highest number of newly approved drugs featuring nanocrystalline APIs during this period. The commercial value of this technology is further enhanced by the relatively short time required for clinical approval and its ability to transform old drugs into new patentable drug products with improved therapeutic profiles and greater patient benefits [3]. The first patent application for Emend^®^ was filed in 1990, with the product reaching the market in 2000. Therefore, compared to other nanotechnology-based platforms, a significant number of nanocrystalline pharmaceutical products have been successfully developed and brought to market within a limited timeframe. NCs, or nanosuspensions, are a relatively underrecognized yet highly versatile platform with broad application potential. Due to their advanced particle engineering capabilities, they can be incorporated into advanced drug delivery systems. In recent years, we have explored the application of nanonization technology to enhance the intranasal delivery of poorly water-soluble molecules to the brain [4,5]. For over a decade, we have been investigating the intranasal route as an alternative approach for delivering molecules (monoclonal antibody, fluorescent probe, oxcarbazepine, etc.) to the brain [6,7,8]. This administration route has garnered increasing interest among researchers due to its potential to bypass the blood–brain barrier (BBB) and enable the direct transport of molecules to the central nervous system (CNS), as extensively discussed in recent interesting reviews [9,10]. The intranasal route is a non-invasive method of drug administration that enables dose reduction and minimizes peripheral side effects. Although intranasal delivery is characterized by numerous advantages, it also presents several limitations, many of which we have encountered during experimental investigations. One of the main limitations is the small volume that can be administered into the nasal cavity. This constraint poses challenges in formulating polymeric or lipid-based nanocarriers at sufficiently high concentrations while maintaining properties (viscosity, homogeneity, etc.) compatible with intranasal dosing. In contrast, NCs, being ‘carrier-free’ systems composed entirely of the APIs and a stabilizing agent, are capable of maintaining a potent therapeutic concentration in small dosing volumes, particularly advantageous for intranasal application. Although NCs have not yet been extensively investigated for intranasal administration, some authors have demonstrated their feasibility in achieving brain delivery via this route. These preliminary findings support the potential of NC-based formulations for effective nose-to-brain transport, warranting further exploration in this area. For example, in vivo pharmacokinetic studies demonstrated the superior brain-targeting efficiency of intranasally administered paeoniflorin NC (PA-NC) compared to intravenous administration in rats. These findings indicate that intranasal delivery facilitates enhanced drug transport, allowing direct access to the brain while minimizing systemic circulation. Notably, PA-NC achieved drug targeting efficiency (DTE%) and direct transport percentage (DTP%) values of 416.60% and 76.50%, respectively, underscoring their potential as an effective strategy for CNS drug delivery [11]. Darweesh and colleagues demonstrated that tadalafil NCs achieved higher concentrations in the brain after intranasal administration in rats compared to the free drug, confirming that the presence of tadalafil NCs significantly improved the pharmacokinetic parameters of the drug compared to the pure one [12].

Current research efforts are increasingly directed toward engineering and functionalizing NC surfaces to support different in vivo applications, including monitoring disease progression and the enhancement of the therapeutic efficacy of existing treatments [13]. The use of positively charged surface modifiers has emerged as particularly advantageous. These agents not only provide electrostatic stabilization by generating repulsive forces that prevent aggregation but also impart functional properties that can enhance mucoadhesion, cellular uptake, and targeted delivery. Overall, surface functionalization with cationic agents represents an advantageous step in the design of NC formulations intended for nose-to-brain delivery, which requires a prolonged residence time in the nasal environment to improve direct drug transport to the brain. Resveratrol (RSV), belonging to Class II of the BCS and acting as an antioxidant and a neuroprotective agent, was selected as a model drug for its antioxidant and neuroprotective effects [14,15,16]. To the best of our knowledge, the functionalization of RSV NCs for brain delivery through the intranasal route has not been investigated yet. With this in mind, the aim of this study was the design of RSV NCs by a Quality by Design approach. The RSV NCs were developed using the design of experiment (DoE) methodology by investigating the effect of the stabilizer concentration % *w*/*v*, the solvent to antisolvent ratio (*v*/*v*), and the stabilizer type (Pluronic^®^ F127 (P127); Tween^®^ 80), indicated as X_1_, X_2_, and X_3,_ respectively, on the NC mean size, polydispersity (PDI), and zeta potential (ZP) codified in turn as Y_1_, Y_2_, and Y_3_.

In order to improve the RSV NC’s stability and promote its intranasal applicability, the optimized nanosuspension was further surface modified with the cationic poly-L-arginine hydrochloride (pArg) homopolymer. Thus, the optimized naked and modified nanosuspensions (labeled as RSV_NC and pArg_RSV NC, respectively) were subjected to a deep physico-chemical and technological characterization to assess their mucoadhesive properties; behavior in artificial cerebrospinal fluid (aCSF); thermotropic features; crystallinity after nanonization; and solubility and stability after being in storage for up to 3 months. The scavenging activity % was also measured by the DPPH assay. Finally, the in vitro investigation was performed on human microglial (HMC3) cells, which are the primary immunocompetent cells in the brain. This preliminary study aimed to assess the safety of nanonized formulations with respect to RSV.

## 2. Materials and Methods

### 2.1. Materials

*Trans*-resveratrol (purity > 95%) was a kindly gifted product. Ethanol, P127, Tween^®^ 80, poly-L-arginine hydrochloride (pArg; mol wt 5000–15,000), mucin from porcine stomach type II, 3-(4,5-dimethylthiazol-2-yl)-2,5-diphenyltetrazolium bromide (MTT) salt, phosphate-buffered saline (PBS) (10X), MEM non-essential amino acids solution (100X), sodium pyruvate (100 mM), and dimethyl sulfoxide (DMSO) were purchased from Sigma-Aldrich (Merck Life Science S.r.l., Milan, Italy). aCSF was prepared by using magnesium chloride anhydrous ≥ 98%, monobasic sodium phosphate heptahydrate, and disodium phosphate monohydrate, all Sigma-Aldrich (Merck Life Science S.r.l., Milan, Italy) products. Sodium chloride was purchased from VWR Chemicals (VWR Chemicals, Radnor, PA, USA), potassium chloride was purchased from Fluka Chemika (Fluka Chemie AG, Buchs, Switzerland), and calcium chloride 98% was purchased from Riedel-de Haen (Riedel-de Haën, Seelze, Germany) (). HMC3 cells (ATCC^®^ CRL-3304™, American Type Culture Collection, Manassas, VA, USA), Eagle’s Minimum Essential Medium (EMEM), fetal bovine serum (FBS), trypsin-EDTA solution, and penicillin and streptomycin solutions were purchased from American Type Culture Collection (ATCC, Manassas, VA, USA). C-Chip disposable hemocytometers were supplied by Li StarFish S.r.l. (Li StarFish S.r.l., Cernusco sul Naviglio, MI, Italy). 2,2-Diphenyl-1-picrylhydrazyl (DPPH) and 6-hydroxy-2,5,7,8-tetramethylchroman-2-carboxylic acid (Trolox) were purchased from Sigma-Aldrich (Sigma-Aldrich, St. Louis, MO, USA).).

### 2.2. Experimental Design

The formulation of RSV NC was designed using Design-Expert software (version 13.0.0, Stat-Ease Inc., Minneapolis, MN, USA). An I-Optimal design was employed to determine the experimental space and identify the optimal levels for each variable. The experimental setup (Table 1) included three factors at varying levels, resulting in 17 experimental runs aimed at optimizing the selected variables. These included two numerical factors—stabilizer concentration (X_1_) and solvent-to-antisolvent (S/AS) ratio (X_2_)—as well as one categorical factor (X_3_), the type of stabilizer (P127 or Tween^®^ 80). The impact of these variables on the particle size (Y_1_), PDI (Y_2_), and ZP (Y_3_) of the NC was evaluated. Supplementary Table S1 provides the specific combination of independent variables for each experiment. Data were statistically analyzed using ANOVA as implemented in the software.

### 2.3. Preparation of Naked Nanocrystals by Sonoprecipitation Technique

RSV NCs were produced using the sonoprecipitation technique, which combines precipitation with ultrasonication via a probe sonicator (Branson Sonifier 450, Marshall Scientific, Hampton, NY, USA). The solvent phase consisted of 12 mg of RSV dissolved in 1.2 mL ethanol. The antisolvent phase was prepared by dissolving varying concentrations of surfactant according to the experimental design (Table 1) in deionized water. The solvent was then introduced into the cooled antisolvent (maintained at ~1 °C using an ice-water bath) under magnetic stirring at 650 rpm. This was immediately followed by two cycles of high-intensity sonication using an ultrasonic probe operating at 400 W power input and 10–100% amplitude for a total duration of 10 min, at a constant frequency. The spontaneous formation of RSV NC was indicated by the development of turbidity in the solution. The resulting nanosuspension was left under a fume hood overnight to allow complete evaporation of residual ethanol.

### 2.4. Particle Size, Polydispersity, and Zeta Potential Analysis

Particle size, PDI, and ZP of both naked and functionalized RSV NCs were measured using photon correlation spectroscopy (PCS) with a Zetasizer Nano S90 (Malvern Instruments, Malvern, UK). Measurements were conducted at a fixed detection angle of 90°, at 25 °C, using a 4 mW He–Ne laser operating at a wavelength of 633 nm. All measurements were performed after dilution in water (1:10) in triplicate, and the results are reported as mean ± standard deviation (SD).

### 2.5. Naked Nanocrystal Optimization

Optimization of RSV NC was carried out using the desirability function available in the Design-Expert software (version 13.0.0, Stat-Ease Inc., Minneapolis, MN, USA). This function identifies the set of experimental conditions that yield the most favorable response outcomes. The aim of the optimization process is to determine the optimal factor settings based on predefined goals for each response, which may include maximizing, minimizing, or targeting specific values. Alongside these goals, acceptable upper and lower limits must also be defined. The software then automatically evaluates combinations of the independent variable levels to determine the optimal conditions within the experimental design space. The resulting desirability values range from zero (completely undesirable) to one (fully desirable).

### 2.6. Scanning Electron Microscopy (SEM) of Optimized RSV NC

The optimized nanosuspension was subjected to morphology investigation by Scanning Electron Microscopy (field emission gun SEM Zeiss, Oberkochen, Germany). Specifically, RSV NC (0.01 g/mL) was analyzed after 1:5 dilution. A drop of nanosuspension was slid onto a silicon wafer to perform the measurements using the InLens detector.

### 2.7. Polycation–Functionalization of Optimized RSV NC

Naked RSV NCs were modified by surface functionalization with poly-L-arginine hydrochloride (pArg) to obtain positively charged NC (pArgRSV_NC). In particular, pArg solution in distilled water (0.05% *w*/*v*) was added under agitation (250 rpm) to RSV NC suspension (1:5 *v*/*v*) at 25 °C for 24 h.

### 2.8. Physico-Chemical and Technological Characterization of Naked and Polycation-Functionalized RSV NC

#### 2.8.1. Lyophilization

RSV_NC and pArgRSV_NC were freeze-dried to obtain dry powders for further technological evaluations. The samples were initially frozen at −56 °C for 24 h using a deep freezer. Subsequently, freeze-drying was carried out for 24 h at 0.1 mbar using a BUCHI Lyovapor™ L-200 Freeze Dryer (Fisher Scientific, Rodano MI, Italy) to produce the dry powders. The resulting lyophilized nanosuspensions were then characterized through drug content analysis, saturated solubility testing, thermal analysis, and Fourier transform infrared (FT–IR) spectroscopy.

#### 2.8.2. Drug Content

The drug content of the lyophilized RSV_NC and pArgRSV_NC was determined by dissolving a precisely weighed portion of the optimized freeze-dried samples in 10 mL of ethanol, followed by shaking at room temperature. The amount of drug was measured using a UV–VIS spectrophotometer (UH5300 UV-Visible Double-Beam Spectrophotometer; Hitachi Europe, Milan, Italy) at a wavelength of 306 nm. The calibration curve for RSV quantification was linear between 1 and 50 μg/mL, with an R^2^ value of 0.999. Measurements were conducted in triplicate, and the mean values and standard deviations (SDs) were calculated.

#### 2.8.3. Saturation Solubility

The saturation solubility of RSV, RSV_NC, and pArgRSV_NC was determined using the solvent saturation method. Briefly, an excess amount of each powder was suspended in vials containing 2 mL of deionized water. The vials were shaken at 25 °C for 24 h, followed by an additional 24 h period to allow the system to reach equilibrium. Samples were centrifuged at 10,000 rpm for 30 min at 25 °C. The supernatants were collected and analyzed for RSV content using a UV–VIS spectrophotometer (UH5300 UV-Visible Double-Beam Spectrophotometer; Hitachi Europe, Milan, Italy) at a wavelength of 306 nm. Quantification was performed using a standard calibration curve prepared in ethanol–water (20:80) with a linear range of 0.9–15 μg/mL and an R^2^ value of 0.997. All experiments were conducted in triplicate, and results are reported as mean ± standard deviation (SD).

#### 2.8.4. Stability and Interaction of Naked and Surface-Modified Nanosuspensions in the Presence of Nasal Mucus Component

RSV NCs (RSV_NC and pArgRSV_NC) were incubated with a mucin dispersion (1:1 *v*/*v*) prepared in simulated nasal fluid (SNF: 2.192 g NaCl, 0.145 g CaCl_2_, and 0.745 g KCl dissolved in 250 mL of double-distilled water; pH 5) at 37 °C to assess the stability and interaction between the NC and mucin. The mean particle size, PDI, and ZP of the NC/mucin mixtures were determined by PCS at specified time points (0, 1, and 3 h). Additionally, the interaction was evaluated using a turbidimetric assay, where absorbance measurements at 650 nm were taken via UV–Vis spectrophotometry for both native mucin and RSV NC–mucin dispersions after incubation periods of 0, 30 min, 1 h, and 24 h.

#### 2.8.5. Mucoadhesive Binding

The mucoadhesive properties of RSV_NC and pArgRSV_NC were assessed based on their interaction with negatively charged mucin. In brief, equal volumes of mucin solution (0.1% *w*/*v* in SNF) and the nanosuspensions of RSV_NC or pArgRSV_NC were mixed and stirred for 15 min at room temperature then incubated at 37 °C for 1 and 3 h. Following incubation, the mixtures were centrifuged at 12,000 rpm for 1 h at 25 °C using a ThermoScientific™ SL16R centrifuge (ThermoFisher Scientific, Waltham, MA, USA). The concentration of free mucin remaining in the supernatant was measured at 260 nm using a UV-VIS spectrophotometer (UH5300 UV-Visible Double-Beam Spectrophotometer; Hitachi Europe, Milan, Italy). The mucin calibration curve was linear over the range of 93.75–1000 µg/mL (R^2^ = 0.998). The mucin-binding efficiency (BE%), which reflects the mucoadhesive strength of the NC, was calculated using Equation (1):

(1) $$Mucin\;Binding\;Efficiency\;\%=\frac{total\;amount\;of\;mucin-free\;amount\;of\;mucin}{total\;amount\;of\;mucin}\times 100$$

#### 2.8.6. Naked and Polycation-Functionalized RSV NC Behavior in Artificial Cerebrospinal Fluid

The mean particle size and PDI of RSV_NC and pArgRSV_NC were assessed following incubation in aCSF. Both naked and surface-functionalized nanosuspensions were combined with freshly prepared aCSF, which consisted of an equal mixture of solution A and solution B. Solution A contained NaCl (8.66 g), KCl (0.224 g), CaCl_2_·2H_2_O (0.206 g), and MgCl_2_·6H_2_O (0.163 g) dissolved in 500 mL of deionized water, while solution B consisted of Na_2_HPO_4_·7H_2_O (0.214 g) and NaH_2_PO_4_·H_2_O (0.027 g) dissolved in 500 mL of deionized water [5]. Aliquots were incubated at 37 °C and analyzed at predetermined time points (0, 30 min, 1 h, and 24 h) using PCS to monitor potential aggregation. Statistical significance was assessed using Dunnett’s multiple comparisons test, with differences considered significant at *p* < 0.05.

#### 2.8.7. Differential Scanning Calorimetry

Thermal analyses were performed on the pure drug, P127, pArg, physical mixtures (p.m.) of pArg, P127, and RSV, as well as on freeze-dried RSV_NC and pArg_RSV NC. The analyses were conducted using a Mettler Toledo DSC 1 STARe system equipped with a PolyScience temperature controller (PolyScience, Niles, IL, USA). The detection was achieved using an HSS8 high-sensitivity sensor (comprising 120 gold–gold/palladium–palladium thermocouples) along with a ceramic sensor (Mettler Full Range; FRS5) containing 56 thermocouples. Calibration of the calorimetric system for both temperature and enthalpy was performed using indium, following the standard DSC1 Mettler TA STARe protocol. Approximately 3 mg of each sample was accurately weighed, placed in an aluminum crucible, and sealed with an aluminum lid using a sealing machine. Thermograms were recorded at a heating rate of 5 °C/min across a temperature range of 25–300 °C, followed by cooling scans performed at 10 °C/min. Thermal parameters, including onset temperature, melting point, and endothermic/exothermic enthalpy changes, were extracted using the Mettler STARe Evaluation software (version 13.00) installed on an Optiplex 3020 Dell workstation.

#### 2.8.8. Fourier Transform Infrared Spectroscopy (FTIR)

Attenuated total reflection Fourier transform infrared (ATR-FTIR) spectroscopy was conducted using a Thermo Fisher Scientific Nicolet iS50 FTIR spectrometer to analyze freeze-dried RSV_NC and pArgRSV_NC samples, raw materials (RSV, P127, and pArg), and physical mixtures (p.m.) containing equal weights of RSV and P127, as well as RSV, P127, and pArg. For each sample, 64 scans were recorded at room temperature across the spectral range of 4000–600 cm^−1^ with a resolution of 4 cm^−1^. Background absorption was subtracted prior to each measurement.

#### 2.8.9. Stability Study

Stability studies were conducted on RSV_NC and pArgRSV_NC stored at 5 °C for up to three months. Measurements were taken every 7 days during the first month, and then every 14 days throughout the second and third months. Stability was evaluated based on the mean particle size, PDI, and ZP of the NC.

#### 2.8.10. DPPH Assay

After, physico-chemical characterization the free radical scavenging activity of RSV_NC and pArgRSV_NC was evaluated using the DPPH (2,2-75 diphenyl-1-picrylhydrazyl) assay following the procedure of Consoli et al. [17]. RSV in lyophilized NC form was tested after resuspension in water (22mM RSV concentration) and compared with the same concentration of RSV in ethanol. The test was also conducted for the NC samples used for the in vitro study at concentrations of RSV of 25μM and 50 μM to compare the activity with the same concentrations of raw RSV. For this test a reaction mixtures contained 86 μM of DPPH solubilized in ethanol was prepared and added to the samples. After 10 min at room temperature the absorbance at λ= 517 nm was measured in a microplate reader (Biotek Synergy-HT, Winooski, VT, USA).

### 2.9. Propagation, Maintenance, and Analysis of Human Microglia (HMC3) Cell Viability

HMC3 cells were cultured in a complete medium consisting of EMEM, FBS (10%), streptomycin (0.3 mg·mL^−1^) and penicillin (50 IU mL^−1^), sodium pyruvate (1 mM), GlutaMAX™ (1 mM), and MEM non-essential amino acids solution (1x). Cells were cultured in 25 or 75 cm^2^ polystyrene culture flasks, maintained in a humidified environment (37 °C, 95% O_2_, 5% CO_2_), and passaged every 3–5 days based on the confluence of the cells. The day prior to the treatment, trypsin-EDTA solution was used to detach the HMC3 cells, a C-Chip was employed for cell counting, and cells were plated in 96-well plates (22.5 × 10^3^ cells/well). The following day, cells were treated with RSV, pArgRSV_NC, or RSV_NC at 25 and 50 µM concentrations and incubated for a total of 24 h in a humidified environment. The viability of HMC3 cells was measured through the 3-(4,5-Dimethylthiazol-2-yl)-2,5-Diphenyltetrazolium Bromide (MTT) method, as previously described [18]. At the end of the assay, the absorbance at 569 nm was read using a Synergy H1 Hybrid Multi-Mode Microplate Reader (Biotek, Shoreline, WA, USA). Data are the means of two independent experiments (*n* = 8). Values were normalized with respect to untreated control HMC3 cells and are expressed as the percent variation in cell viability.

### 2.10. Statistical Analysis

Statistical analysis was performed using Prism (version 9.5.0. GraphPad Software, Inc., La Jolla, CA, USA). For the statistical analysis, we used one-way analysis of variance (ANOVA), followed by Dunnett’s multiple comparisons test for the analysis of RSV saturation solubility test. We used a two-way ANOVA followed by Tukey’s multiple comparisons test for the analysis of NC mean size and ZP for mucoadhesive evaluation. We used two-way ANOVA followed by Dunnett’s multiple comparisons test for the analysis of NC turbidimetry for mucoadhesive evaluation. We used one-way ANOVA followed by Dunnett’s multiple comparisons test for the analysis of NC mean size after incubation in aCSF for nanosuspensions behavior in artificial cerebrospinal fluid. We used two-way ANOVA followed by Šídák’s and Dunnett’s multiple comparisons test for the radical scavenging activity assay.

In the case of cell viability experiments, statistical analysis was performed by using Graphpad Prism software (version 8.0, Graphpad software, San Diego, CA, USA). One-way ANOVA followed by Tukey’s post hoc test were used for multiple comparisons. Only two-tailed *p* < 0.05 was considered statistically significant. Data were reported as mean ± SD.

For the statistical analysis of the diameter distribution SEM, OriginPro (version 2019, OriginLab Corporation, Northampton, MA, USA) was used, and the data were fitted using a Gaussian function.

## 3. Results and Discussion

### 3.1. Knowledge Space and Experimental Domain Construction

The QbD approach is part of pharmaceutical development at the industrial level. Currently, QbD is also employed in nanomedicine research within the academic context to promote the translation of basic research into commercially available therapeutic products. DoE is a tool of QbD in which the values of all the variables are modified simultaneously in each experiment, thus a reduced number of experiments can be run to correlate the interaction between variables and optimize the process. DoE exploits statistical algorithms, mathematical models and equations, and prediction tools to optimize process or product conditions and obtain desired target specification [19]. In this work, the DoE was applied to design a formulation based on RSV NCs conceived for brain delivery after intranasal administration. Firstly, the critical process parameters (CPPs) and critical material attributes (CMAs) that can affect the product outcome were identified. As mentioned, RSV NCs were conceived for nose-to-brain delivery application, thus, CQAs of RSV NCs, such as their mean size, PDI, and surface charge, are of utmost importance and must fit specific criteria. As reported in our previous study, in order to obtain direct delivery to the brain, particles should possess a diameter smaller than that of olfactory axons (100–700 nm) to be intracellularly transported via the olfactory neural pathway [4]. Furthermore, the particles’ surface properties may affect their behavior after nasal administration [5]. Regarding the CMAs and CPPs, once the preparation method was established, a preliminary investigation was performed to select the factors that must be kept as constant and those critical to build the experimental domain. The stabilizer plays a key role in NCs’ formation and size since it provides their physical stability by reducing their free surface energy, inhibiting their aggregation and Ostwald ripening, and changing the crystalline shape of nanosized particles during preparation and storage [20]. Moreover, not all the stabilizers are applicable for intranasal use because their local tolerance is often unsuitable and, therefore, has to be carefully evaluated. Based on these considerations, the concentration and the type of stabilizer were selected as independent variables and investigated using DoE. Specifically, P127 was selected based on its non-toxic properties and ability to interact with hydrophobic surfaces and biological membranes [21], while Tween^®^ 80, as reported in the literature, was proven to be a promising excipient to increase drug concentrations in both plasma and the brain via intranasal routes [22]. The last independent variable considered was the solvent to antisolvent ratio (S/A), since this parameter may affect the degree of supersaturation and thus the nucleation process during NC formation. Therefore, in accordance with the literature, the effect of different S/A phase ratios (i.e., 1:1, 1:2, 1:5) was evaluated for RSV NCs’ mean size, PDI, and ZP [4]. In the pursuit of a robust experimental design for our investigation, which involves two numeric factors and one categorical factor, the I-Optimal design strategy was selected over other approaches for its flexibility, precision, and adaptability to complex factor types and experimental constraints. The I-Optimal design accommodates both numerical and categorical factors within a single, integrated design structure. The I-optimality criterion minimizes the average variance of the predicted response across the entire design space [23]. This is a crucial advantage when the primary objective is to develop a highly accurate predictive model. For our study, achieving the highest possible precision across the range of numerical factor variations and categorical factor levels is paramount for drawing robust conclusions and ensuring generalizability. I-Optimal designs provide unparalleled flexibility in specifying the exact number of experimental runs, allowing researchers to select the minimum number of runs necessary to achieve the desired model estimability and precision.

#### 3.1.1. Outcome of Independent Variables for RSV NC Mean Size

RSV NCs were prepared by the solvent–antisolvent method according to DoE (Supplementary Table S1). A total of 17 runs were carried out, obtaining a mean size in the range of 26.7 ± 12.1 – 1300 ± 54 nm and exhibiting a significant quadratic model (*F*-value = 24.95; R^2^ = 0.9615) with a similar Adjusted (0.9229) and Predicted R^2^ (0.8217), which demonstrates a good fit between the experimental results and the values predicted by the polynomial equation describing the model (Equation (2)):

(2) $$Mean\;size=64.1255+\left(-305.907\right)\times A+50.3232\times B+132.645\times C+\left(-21.0458\right)\times AB\;+(-99.0612)\times AC+147.244\times BC+325.26\times A^{2}+144.82\times B^{2}$$

**Figure 1:**
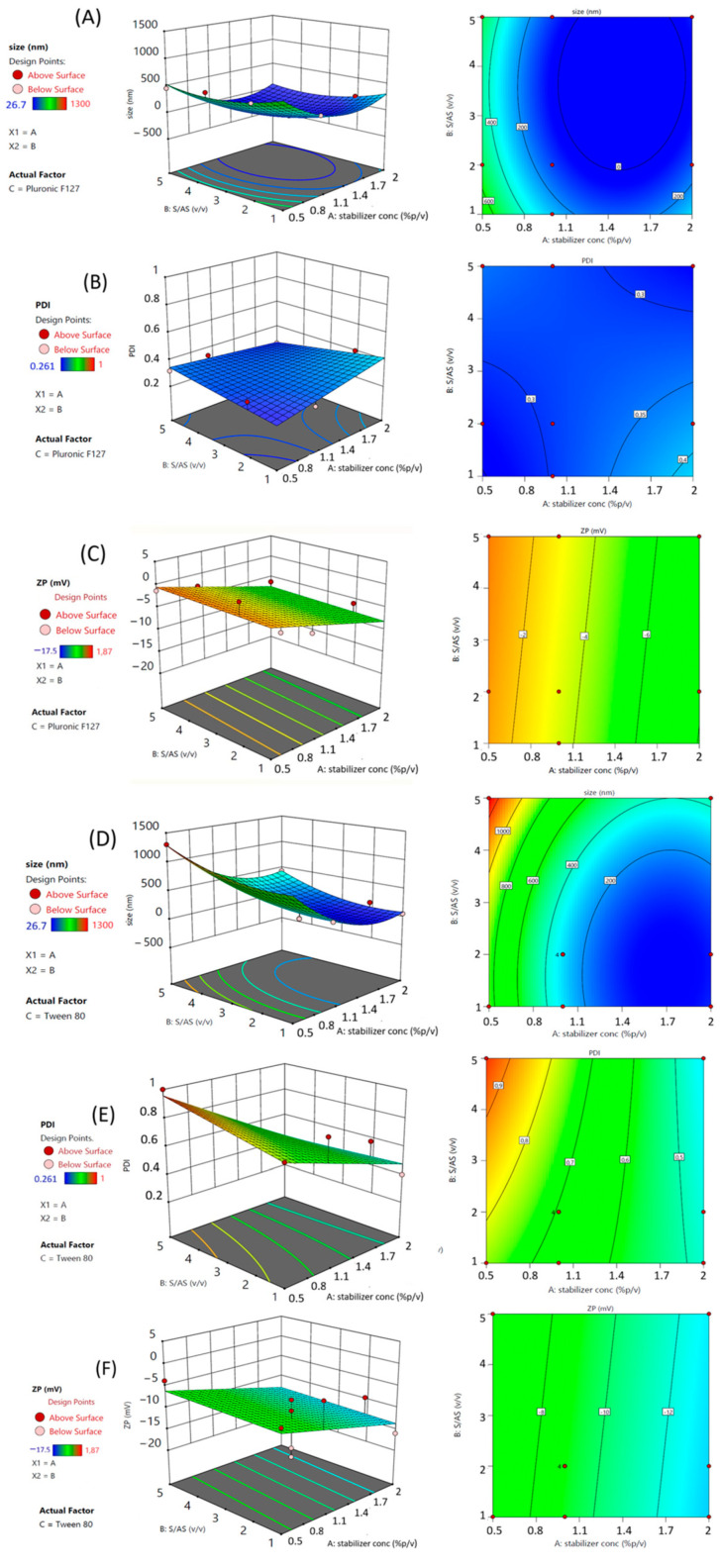
Three-dimensional surface and contour plot of the effect of S/AS ratio versus stabilizer concentration (*w*/*v*%) on RSV NC mean size (**A**), PDI (**B**), and ZP (**C**) using Pluronic F127. Three-dimensional surface and contour plot of the effect of S/AS ratio versus stabilizer concentration (*w*/*v*%) on RSV NC mean size (**D**), PDI (**E**), and ZP (**F**) using Tween80.

Two-dimensional and three-dimensional contour maps (Figure 1A,D) allow for the visualization of results across a broad spectrum of experimental conditions (knowledge space) and help identify the range of parameters suitable for meeting desired target criteria (design space).

“A”, “C”, “AC”, “BC”, and “A^2^” were significant model terms. Factor “A” refers to the stabilizer concentration, followed by the stabilizer type “C”, the interaction between the stabilizer concentration and type “AC”, and the interaction between the S/AS ratio and the stabilizer type “BC”. As reported, all the model variables affected the mean size response as individual, interaction, or quadratic terms.

The coefficient estimate for each significant term indicates the expected change in the response for every one-unit change in the factor, assuming all other factors remain constant. Factor A showed a negative coefficient (−305.91), indicating that as the surfactant concentration increases, the NC size decreases, suggesting its primary role in limiting crystal growth. Term C (stabilizer type) showed a positive coefficient (+132.645), indicating that, when numerical factors are at their center points, using Tween 80 results in significantly larger NCs compared to using P127.

A significant effect was observed for the interaction term “AC”. This significant negative interaction term (−99.0612) indicates that the effect of the stabilizer concentration on the NC size is dependent on the stabilizer type. Specifically, the reducing effect of increasing the stabilizer concentration on the NC size is more pronounced when Tween 80 is used compared to P127. Even the interaction term BC was found to be significant. The positive interaction term (+147.244) signifies that the effect of the S/AS ratio on the NC size also varies with the stabilizer type. The positive effect of increasing the S/AS ratio (increasing size) is considerably more pronounced when Tween 80 is used compared to P127. Finally, the quadratic term A^2^ was found to be significant (+325.26). The positive quadratic term for the stabilizer concentration indicates a non-linear relationship, suggesting a U-shaped or convex response. While the main effect of A is negative, this positive quadratic term implies that beyond a certain concentration, the rate of the size reduction might decrease, or even reverse, leading to an increase in size at very high concentrations, assuming other factors are held constant. If the stabilizer concentration is too low, drug particles cannot be adequately coated. However, nanosuspension stability does not increase linearly with higher stabilizer levels [24,25]. Excessive stabilizer concentrations can cause Ostwald ripening, reducing stability over time. Additionally, when amphiphilic stabilizers exceed their critical micelle concentration (CMC), micelles may form. These micelles can compete with the stabilizer adsorption on the particle surface, leading to the instability of the NC and an increase in particle size [26]. Therefore, selecting an appropriate stabilizer concentration is essential. This conclusion is supported by the correlation coefficient magnitude, which reflects the strength of this relationship [27]. In this case, term “A” presents the highest magnitude, meaning that this factor exerted the maximum weight on this response. This suggests an optimal stabilizer concentration might exist within or near the experimental range. These results reveal that the stabilizer concentration is the most dominant factor in reducing the NC size.

#### 3.1.2. Outcome of Independent Variables on RSV NC Polydispersity

PDI, a parameter used to measure the width of the particle distribution, was found in the range between 0.26 ± 0.03 and 1.00 ± 0.23 for the 17 runs and exhibits a significant 2FI model (*F*-value = 16.76; R^2^ = 0.9096) with a similar Adjusted (0.8553) and Predicted R^2^ (0.7225), indicating congruity between the experimental and the predicted values by the polynomial equation governing this model (Equation (3)):

(3) $$PDI=0.487615+\left(-0.0889565\right)\times A+0.0123878\times B+0.16886\times C+\left(-0.0614504\right)\times AB\;+(-0.112486)\times AC+0.0254167\times BC$$

“A”, “C”, and “AC” are significant model terms. Figure 1B,E illustrates the combined influence of the factors as revealed through contour and three-dimensional response surface plots. The contour and response surface plots indicate that the PDI of the nanosuspensions was strongly dependent on the effect of the investigated independent variables, as both homogeneous and highly polydisperse samples were obtained. The concentration of the stabilizer and the type of stabilizer played a key role in this response as individual and interaction terms (2FI model). Factor “A” (stabilizer concentration) showed a significant negative coefficient (−0.0889565), indicating that increasing the stabilizer concentration linearly decreased the PDI. This suggests that higher stabilizer concentrations promote a narrower and more uniform NC size distribution. The positive coefficient (+0.16886) found for factor C (stabilizer type) indicates that, when numerical factors are at their center points, using Tween 80 results in a significantly higher PDI compared with P127, suggesting that P127 promotes a more uniform size distribution. The interaction term AC (stabilizer concentration, stabilizer type) showed a significant negative coefficient (−0.112486), indicating that the effect of the stabilizer concentration on PDI depends on the stabilizer type. The PDI reducing effect of increasing the stabilizer concentration was considerably more pronounced when Tween 80 was used compared to P127. Our findings clearly demonstrate that the stabilizer concentration is the main factor for achieving a narrow size distribution (lower PDI). Furthermore, the type of stabilizer plays a critical role, with P127 generally leading to more uniform particles than Tween 80 under similar conditions. The significant interaction terms highlight that the optimal settings for numerical factors for achieving low PDI are dependent on the chosen stabilizer type. These findings are crucial for fine-tuning the preparation parameters to control the homogeneity of the NC population, which is essential for consistent performance in various applications.

As mentioned, NCs are simple drug particles surrounded by a stabilizer, and the latter component plays a crucial role in NC formation and stability. Due to their high surface energy, NCs tend to aggregate, leading to physical instability and particle growth. To counteract this, stabilizers are used to enhance both chemical and physical stability by adsorbing onto the NC surface and reducing interfacial tension. Different types of stabilizers can be used, ranging from steric stabilizers, surfactants, to functional stabilizers. The nature of the stabilizer determines its mechanism of action. Ionic surfactants stabilize nanosuspensions through electrostatic repulsion among drug NCs. Specifically, the adsorption of the ionic stabilizer onto the drug surface creates a double electric layer from its hydrophilic portion, generating a charge around the drug particles. When these particles approach each other, the like-charged layers repel, preventing aggregation and keeping the particles separated [28]. Polymers and nonionic surfactants like P127 and Tween 80 stabilize nanosuspensions by creating spatial barriers that act as steric stabilizers. These stabilizers adsorb onto the surfaces of hydrophobic drug NCs, and their long hydrophilic polymer chains restrict the movement of the drug particles, helping to keep them separated. Unlike ionic surfactants, the stability provided by steric hindrance is not affected by ionic charges, which also allows for the surface modification of NCs with coating materials without causing formulation problems [29]. Another crucial factor influencing a stabilizer’s effectiveness is its molecular weight (MW). Stabilizers with longer chains can more effectively create spatial repulsion, thereby preventing particle aggregation [30]. Polymeric stabilizers with an MW below 5000 g/mol (such as Tween 80, which has an MW of approximately 1310 g/mol) often fail to provide sufficient spatial barriers, whereas stabilizers with higher molecular weights (like P127, with an MW around 12,500 g/mol) may cause NC bridging due to their extended chain length [28].

#### 3.1.3. Outcome of Independent Variables on RSV NC Zeta Potential

The effect of the surfactant type, concentration, and S/AS ratio was also investigated on the RSV NC surface charge (Figure 1C,F). All samples show a ZP between −17.5 ± 5.9 and +1.9 ± 3.7 mV ± SD and exhibit a significant linear model (*F* value = 4.78; R^2^ < 0.824) with a similar Adjusted and Predicted R^2^, showing consistency between the experimental and the predicted values by the polynomial equation driving this model (Equation (4)):

(4) ZP=(−7.09409)+(−3.40429)×A+0.351646×B+(−2.78979)×C

“A” and “C” are significant model terms. Factor A (stabilizer concentration) showed a significant negative coefficient (−3.40429), indicating that increasing the stabilizer concentration linearly leads to a decrease in ZP. This suggests that higher stabilizer concentrations reduce the surface charge of the NC, potentially due to increased shielding or adsorption, which could affect colloidal stability. The negative coefficient (−2.78979) found for term C (stabilizer type) indicates that, when numerical factors are at their center points, using Tween 80 results in a lower (more negative) ZP compared to using P127. This suggests that the type of stabilizer has a distinct effect on the surface charge of the NC, with Tween 80 imparting a more negative charge or reducing a positive charge more effectively than P127. These results clearly demonstrate that the stabilizer concentration together with the type of stabilizer are the most impactful factors in modulating ZP. Both stabilizers belong to the non-ionic category; thus, their presence on the NC surface does not confer any charge. In fact, as indicated by the coefficient estimate (−3.40), increasing the surfactant concentration led to the formation of NCs with an almost neutral ZP.

### 3.2. Preliminary Characterization of Optimized RSV NC

After identifying the key factors influencing the process, the subsequent step comprised determining the optimal settings of these variables to achieve the desired outcomes during the “optimization” phase. The Response Surface Methodology (RSM) utilizes the desirability function to optimize multiple responses simultaneously. This approach identifies the operating conditions that yield the most favorable response values based on predefined goals for each factor and response (as shown in Supplementary Table S2). The numerical search algorithm explores the design space to locate regions where all response criteria are satisfied concurrently. In this study, the objective was to minimize both the NC size and PDI to produce uniform particles with a mean diameter suitable for direct intranasal brain delivery, while also targeting minimal ZP values to ensure higher absolute zeta potential, which is critical for preventing particle aggregation.

The formulation suggested by the software with the highest desirability (0.920) was validated experimentally, confirming the reliability of the model used (error % 7.7 for NC mean size). In Table 2 the composition and the final features of the optimized RSV NC are reported.

SEM imaging confirmed that the optimized nanosuspension presented a mean diameter of ~240 nm (Supplementary Figure S1A,B), as previously measured by PCS, and revealed their shape as truncated cubic crystals (Supplementary Figure S1A).

Our results suggest that RSV NCs’ morphology was probably affected by the sonoprecipitation technique used for their preparation, which involves an ultrasonic cavitation. The cavitation is characterized by intense local conditions (vigorous shock waves, high generated shear, high pressure differential), which control and promote particle crystallization and precipitation and could also impact their homogeneity and morphology [31]. This idea is in accordance with Argenziano et al., who prepared RSV NCs with a milling process and obtained, in that case, particles with a size of 445.8 nm ± 35.3, characterized by a rounded morphology [32]. Singh et al. obtained RSV NCs by probe sonication dispersing RSV in an aqueous solution of a stabilizer (TPGS) with co-stabilizers (lecithin and P127) followed by homogenization using an Ultra-Turrax and then sonication and lyophilization with mannitol (5%*w*/*v*) as a cryoprotectant. The lyophilized RSV NC showed a mean size of 110.3 ± 12.5 and appeared with a roughly rod shape [33]. The drug content assay revealed that approximately 80% of RSV was retained in the optimized nanosuspension, suggesting some drug loss during the preparation procedure. With the aim of obtaining a formulation appropriate for intranasal administration, the optimized RSV NCs were further subjected to a surface modification process. The surface modification of drug NCs using polycations can contribute to improved chemical and physical stability, while also enhancing drug delivery to specific organs or cellular compartments. The naked formulation was functionalized with pArg, a biodegradable polyaminoacid belonging to the cell-penetrating peptide family. Considering that the rapid nasal mucociliary clearance could restrain particle permanence in the nasal cavity, an ideal formulation should be retained after administration to promote particle/drug absorption or penetration [5]. Thus, the effect of a polycation layer on RSV NCs was investigated as a potential strategy to enhance the formulation residence time after administration. The ability of arginine-rich polyamino acids to penetrate cells is primarily linked to the presence of guanidinium functional groups, which are known to interact specifically with cell surface components prior to internalization [34]. Studies have demonstrated that pArg can bind to tight junction proteins such as occludin and zonula occludens-1 (ZO-1), thereby enhancing the paracellular transport of hydrophilic compounds. This interaction has been shown to improve the permeability of peptides across the Caco-2 intestinal epithelial model, as well as nasal and ocular mucosal barriers [35]. Naked RSV NCs were coated with pArg through adsorption of the cationic homopolymer in water at room temperature on the NC surface. Solvents with high dielectric constants are generally preferred for achieving effective electrostatic stabilization, as they enable better shielding of the attractive forces between oppositely charged ions. The dielectric constant of water at room temperature is 78.5, serving as an excellent medium for this purpose. In such a solvent, the close approach of electrostatically stabilized particles is restricted due to the entropy associated with the aggregation of counterions within the diffuse double layer [36]. A naked RSV nanosuspension was labeled RSV_NC, while pArgRSV_NC referred to the surface-modified drug particles. The physico-chemical investigation revealed that the surface modification occurred efficiently since pArgRSV_NCs were homogenous with a mean size of 307.2 ± 80.6 nm, a PDI = 0.25 ± 0.08, and a net-positive charge (ZP = +24.2 ± 0.7 mV). The slight increase in the mean particle size observed for the pArgRSV_NC, along with the inversion of the surface charge compared to the naked NC, provides clear evidence of successful surface functionalization. The drug content was also evaluated for the surface-modified nanosuspension obtaining overlapping results with the naked formulation. The slight loss of the drug is due to the preparation method, which is the same for both nanosuspensions, since the coating process used to obtain the pArgRSV_NC is an additional step that follows the sonoprecipitation technique. Both naked and surface-modified nanosuspensions were investigated to evaluate the effect of their surface characteristic on their potential application for nose-to-brain delivery, as detailed in the following sections.

### 3.3. Technological Characterization of Naked and Surface-Modified RSV Nanosuspensions

#### 3.3.1. Saturated Solubility and Preliminary Evaluation of Nanosuspension Stability

**Figure 2:**
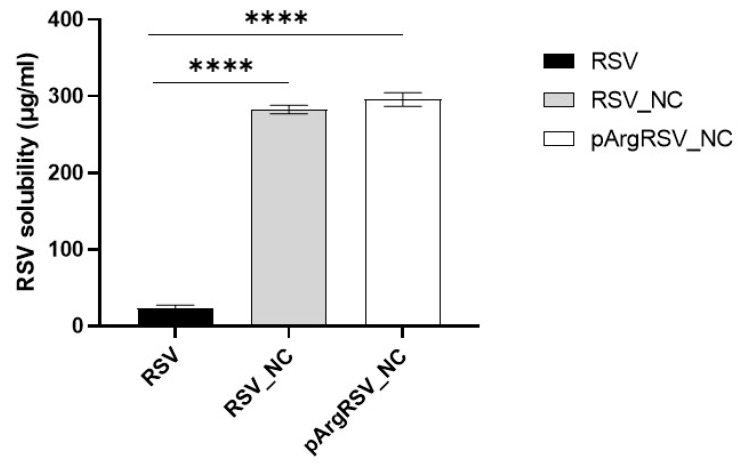
Solubility of pure RSV and freeze-dried RSV_NC or pArgRSV_NC in water. **** *p* < 0.0001 vs. RSV.

The solubility of the RSV, RSV_NC, and pArgRSV_NC in deionized water is shown in Figure 2. 

The solubility of the freeze-dried RSV_NC, pArgRSV_NC, and pure RSV was found to be 282.5 ± 5.5, 295.8 ± 9.0, and 23.5 ±3.8 μg/mL, respectively. RSV has poor solubility in water, RSV_NC showed a ~12-fold enhanced saturation solubility as compared to pure RSV (unprocessed), and a ~13-fold increase was found comparing pArgRSV_NC to the pure drug. Nanonization significantly enhanced the solubility of RSV in water. The influence of pArg in the solubility was slight but interesting. The greater effect on solubility observed with surface-modified NC is likely attributed to the ‘salting in’ phenomenon, where the addition of a charged species such as pArg raises the ionic strength of the solution, thereby enhancing the solubility of hydrophobic molecules like RSV [37]. Although the solubility improvement from the pArg coating was modest relative to naked NCs, it suggests that cationic surface modification may contribute marginally to solubility through ionic interactions.

**Figure 3:**
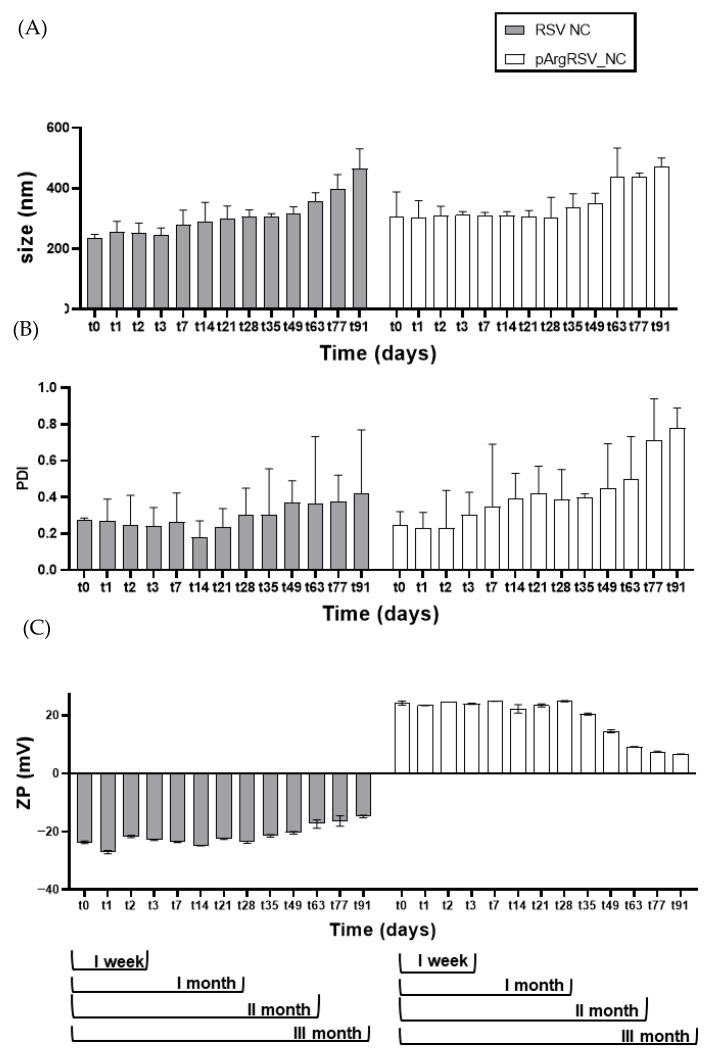
Stability of RSV_NC and pArgRSV_NC at refrigerated temperature up to 3 months in terms of particle size (**A**) and PDI (**B**) and ZP (**C**).

The stability of the RSV_NC and pArgRSV_NC was evaluated at a refrigerated temperature, assessing the variation in the particle size, PDI, and ZP over a period of 3 months. Results showed that both RSV_NC and pArgRSV_NC suffered an increase in particle size after 63 days of storage (Figure 3A) and were roughly homogenous for RSV_NC (PDI ≤ 0.4) over time. Meanwhile aggregation phenomena were registered for pArgRSV_NC since the increase in the particle diameter was accompanied by higher PDI values (PDI = 0.7). A similar trend was found for ZP values (Figure 3B), since RSV_NC maintained an almost constant value (~−23 mV) up to t49, then the ZP started to decrease and reached values equal to −14.80 mV ± 0.03 at t91. The ZP of pArgRSV_NC was constant up to 35 days, confirming the presence of the pArg layer on the NC surface, and then from t49 it started to decrease from ~+24.0 to +6.8 mV ± 0.1 at t91. Stability represents a major challenge in NC technology, as the high surface energy associated with small particles drives a tendency toward particle growth over time in an effort to reduce surface energy. Due to Brownian motion, particles within a nanosuspension are prone to collisions, which may lead to coalescence or adhesion. Such aggregation events result in an increased particle size and a broader particle size distribution [28]. Ostwald ripening refers to the growth of larger crystals resulting from the dissolution of smaller ones, driven by solubility differences between particles of varying sizes. This process results in the dissolution of smaller particles and the progressive enlargement of larger ones. Additionally, particles may undergo flocculation or deflocculation. NC sedimentation is considered acceptable as long as the sedimentation rate is low and the settled particles can be easily redispersed [28]. Overall, our findings suggested that both formulations can be stored as nanosuspensions at 5 °C at least for 49 days, but the stability of the drug in the aqueous suspension over time has to be verified to ensure integrity and the absence of degradation in the same storage condition. The conversion of the RSV_NC and pArgRSV_NC nanosuspension into powder should be investigated in order to preserve samples from physico-chemical modification during storage.

#### 3.3.2. DSC and FTIR Analyses

**Figure 4:**
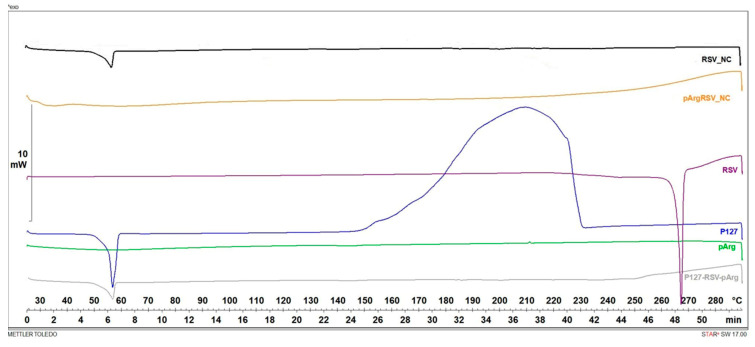
DSC thermograms of the raw materials (RSV; P127; pArg); physical mixture of RSV-P127-pArg, RSV_NC, and pArgRSV_NC.

A thermal analysis was conducted to investigate the thermotropic properties of both naked and polycation-functionalized nanosuspensions. DSC thermograms of the raw materials, their physical mixtures (p.m.), and the freeze-dried forms of RSV_NC and pArgRSV_NC are presented in Figure 4. The thermogram of RSV was typical of a crystalline substance with a sharp endothermic peak at the onset temperature of 257.81 °C. The DSC thermograph of P127 exhibited a sharp endotherm with an onset melting point at 56.71 °C, while pArg (Figure 4) did not show a significant melting peak, confirming its amorphous nature. The physical mixture (p.m.) of P127, RSV, and pArg (Figure 4) displayed an endothermic peak corresponding to P127 at 56.71 °C. The characteristic RSV peak appeared shifted to 249.65 °C and exhibited a broader, less defined shape, likely indicating partial miscibility between the drug and the polymer. This behavior can be attributed to the lower melting point of poloxamers compared to RSV, which may facilitate a degree of interaction or miscibility between the two. The absence of a sharp RSV melting peak supports the hypothesis of reduced crystallinity due to this interaction. The thermogram of RSV_NC revealed the presence of P127, while the sharp endothermic peak of RSV disappeared, probably due to the drug/polymer miscibility as well. Similarly, pArgRSV_NCs were characterized by the absence of an RSV melting peak and also by the disappearance of P127 endothermic peak, probably due to the presence of the pArg coating on the NC surface that masked the presence of the stabilizer layer.

**Figure 5:**
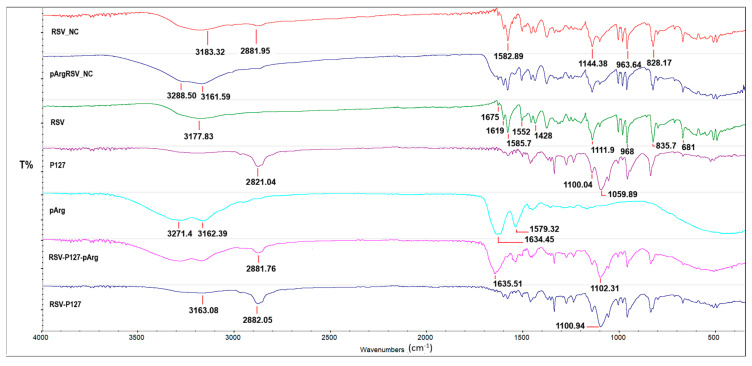
FTIR spectra of the raw materials (RSV; P127; and pArg); physical mixture of RSV-P127-pArg; and RSV-P127, RSV_NC, and pArgRSV_NC with tick marks of the main peaks.

FT-IR analysis is a crucial method for assessing material compatibility within a formulation by scrutinizing its spectrum and comparing it with the spectra of individual components. In the RSV spectrum (Figure 5), the distinctive peak in the 3000 cm^−1^ region, characteristic of phenolic compounds, signifies the phenolic O-H stretching of the H bond [38]. The presence of a peak at 1675 cm^−1^ (C-C aromatic double bond stretching) and at 975 cm^−1^ (trans olefinic band) distinguishes trans-RSV from cis-RSV, and its persistence in NCs confirms the maintenance of RSV in the trans-form, ensuring heightened pharmacological function without isomerization [39]. Peaks in the 700 and 1800 cm^−1^ region are linked to C=C-C aromatic ring stretching bonds (1584.7–1619 cm^−1^ and 1428–1552 cm^−1^) of the stilbene molecule, along with various aromatic out-of-plane C-H (681.5–834.7 cm^−1^) and in-plane (968.3–1211.9 cm^−1^) bending. The uniqueness of the vibrational spectrum as a physical property is evident in both pArgRSV_NC and RSV_NC, underscoring the unaffected crystalline nature of RSV during the nanonization process. Upon examining the spectra of pArg, a double peak in the 3000–3200 band characterizes the N-H stretching of primary amines. Peaks at 3271.4 cm^−1^ and 3162.39 cm^−1^ further characterize primary amines, while the peak at 1634.45 distinguishes primary amines from amines II and III. The peak at 1539.72 belongs to the C=O carboxyl group, typical of zwitterionic amino acids, overlapping with the RSV peak in the spectra of pArgRSV_NC, likely indicating the complete coverage of the NC by the polycation in agreement with DSC findings. Additionally, a distinctive double peak in the pArgRSV_NC spectra differentiates it from RSV_NC. The presence of both pArg bands in the NC suggests an effective covering of the naked NC with the positive amino acid. In p.m., as well as in RSV_NC and pArgRSV_NC, a comparison with the peaks of the pure drug reveals no significant changes, indicating the absence of incompatibility between the drug and excipients. The characteristic peaks of P147 at 2881.04 cm^−1^ correspond to the stretching vibrations of CH-groups, while a broad peak in the 1100–1070 cm^−1^ region (1059.89 cm^−1^ and 1100.09 cm^−1^) corresponds to the stretching vibrations of C-O groups in the ether group. The latter reappears in both the p.m. and the NC, whereas the second peak 2881.04 cm^−1^ clearly appears in the spectra of the mixtures and is more smoothed in the spectra of RSV_NC and pArgRSV_NC. This probably arises due to bonds between RSV and the surfactant in the formation of NCs. Beyond confirming the absence of incompatibility between the materials, the preservation of RSV crystallinity in the formulation suggests that neither the nanonization nor freeze-drying has affected its nature. FTIR and DSC data confirmed that pArg effectively coated the NC surface and masked the signature peaks of P127, implying successful surface modification on naked NCs.

#### 3.3.3. Mucoadhesive Evaluation

**Figure 6:**
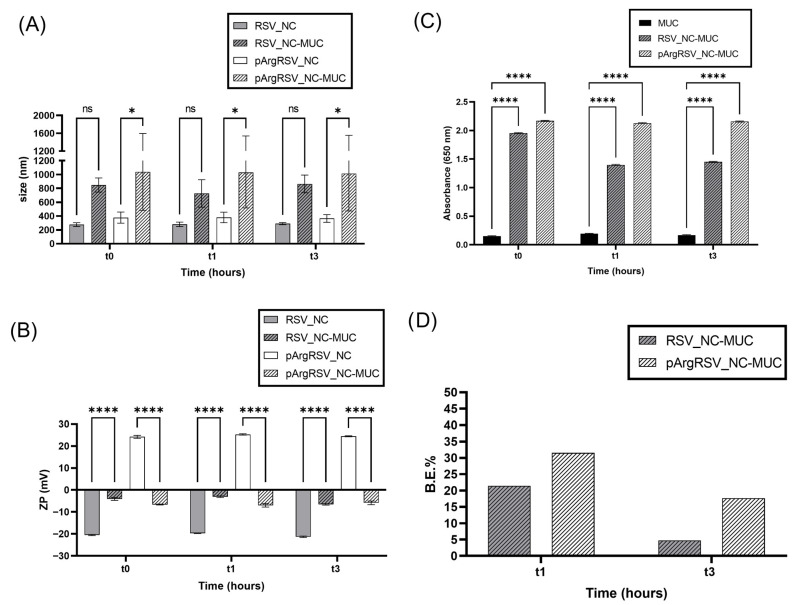
Mean particle size values (**A**) and zeta potential (ZP) values (**B**) of RSV_NC and pArgRSV_NC samples before, 0, and after 1 and 3 h of incubation with mucin (RSV_NC-muc and pArgRSV_NC-muc) at 37 °C, in vitro assessment of samples/mucin interactions at different time points (0, 1, 3 h) by turbidimetric assay at 650 nm (**C**), and binding efficiency % (B.E.%) of RSV_NC and pArgRSV_NC towards mucin after 1 and 3 h of incubation at 37 °C (**D**). Significance was set as *p* > 0.05 (not significant; ns); * *p* ≤ 0.05 for size (nm); **** *p* ≤ 0.0001 for ZP and turbidimetry.

The potential application of RSV_NC and pArgRSV_NC was also evaluated in terms of their mucoadhesive strength and interaction with mucin, the main glycoprotein present at the nasal mucosal level, by using different approaches (Figure 6) to assess the behavior of the formulations in the nasal milieu. Specifically, negatively (naked) and positively (surface-modified) charged nanosuspensions were mixed and incubated with the mucin suspension in the SNF to simulate the nasal milieu at specific time intervals, as well as to analyze the particles size and ZP (Figure 6A,B) compared to the nanosuspension, the turbidimetry (Figure 6C) compared to the native mucin, and the mucin B.E. % (Figure 6D) to measure the interaction strength.

Both nanosuspensions interacted with mucin, as revealed by the increase in the particle size (not significant for RSV_NC) after the incubation with the glycoprotein and the variation in the ZP values (Figure 6A,B), even if higher differences were found for pArgRSV_NC. The turbidity assay (Figure 6C) confirmed the entity of the interaction since the increase in absorbance values for all samples compared to the native mucin indicated the occurrence of aggregation. The evaluation of the mucoadhesive behavior is an interesting and crucial preliminary test when a formulation for nasal administration is conceived. One of the main limitations of intranasal dosing is the rapid mucociliary clearance, a mechanism whose main function is to protect the respiratory system from damage by inhaled substances, which also restricts drug absorption, resulting in a reduced or a lack of efficacy [40]. The mucus layer on the nasal epithelium is made up of roughly 90–95% water, 2–5% mucins, around 1% salts, and varying levels of cellular materials and debris, including DNA, albumin, immunoglobulins, lysozyme, lactoferrin, and lipids [41]. In particular, the highly glycosylated mucins are the main responsible mucus properties. Mucins present a rigid structure with high levels of sialic acid and sulfate residues, which provide a neat negative charge [42]. Various polymers, including natural ones like alginates and gelatin; semisynthetic types such as cellulose derivatives; and synthetic polymers like polyacrylates and polymethacrylates, have been utilized to enhance nasal drug delivery [41]. Mucoadhesion involves a series of processes beginning with the hydration of polymer chains, followed by close contact with mucus and subsequent diffusion and entanglement with mucin fibers. This interaction results in electrostatic attractions, hydrophobic forces, hydrogen bonding, and van der Waals interactions [43]. For instance, chitosan and low-molecular-weight pectins have been demonstrated to extend the nasal formulation’s residence time in the human olfactory region [44]. Additionally, sodium hyaluronate increased the brain delivery of fluorescein-labeled 4 kDa dextran after nasal administration in rats [45]. Nanoparticles with positive charges and hydrophobic surfaces tend to adhere more effectively to mucus due to electrostatic and hydrophobic interactions with mucins. Such interactions with the nasal mucus can prolong the formulation residence time and enhance absorption in the olfactory region, thereby facilitating direct drug delivery to the brain. To address the challenge of rapid nasal mucus clearance and turnover, which limits the retention time of particles, an additional surface modification was evaluated for pArgRSV_NC. This strategy aimed to exploit the synergistic effect between a mucus-penetrating stabilizer (P127) and a cationic agent (pArg), beyond the contribution of the stabilizer alone. Surface-modified NCs (pArg RSV_NC) offer a dual advantage in interacting with the mucus layer. Their positive surface charge enables electrostatic interactions with the negatively charged mucins, thereby promoting mucoadhesion. Simultaneously, the further presence of P127 as a stabilizer facilitates mucus penetration by shielding the NC surface from hydrophobic and electrostatic interactions that would otherwise hinder diffusion. Although certain surface modifications can reduce mucoadhesion, our findings align with the existing literature. Specifically, pArg, a polycationic homopolymer, likely enhanced mucoadhesion, while naked the RSV_NC, stabilized solely by P127, may have exhibited reduced interactions with mucus components, enabling more efficient penetration [46]. For example, Sharma et al. demonstrated that the presence of P127 on PLGA nanoparticles loaded with diazepam or midazolam enhanced the mucus penetration of nanoparticles [47,48]. In both instances, the particles were negatively charged and measured under 200 nm, enabling controlled drug permeation through sheep nasal mucosa, direct transport to the brain, and improved brain targeting efficiency compared to a drug solution following nasal administration in rats [47,48]. Mucoadhesion was also assessed in vitro by determining the B.E.% of RSV_NC and pArgRSV_NC toward mucin (Figure 6D). Our results confirmed, once again, the occurrence of an interaction between both samples with the glycoprotein and revealed more intense mucoadhesive strength for pArgRSV_NC, as showed in Figure 6D, at both investigated time intervals.

#### 3.3.4. RSV Nanosuspension Behavior in Artificial Cerebrospinal Fluid

Drug delivery to the brain remains a major challenge due to the presence of the BBB, which limits the entry of foreign substances and the complexity of the brain microenvironment, which comprises the extracellular matrix, cellular components, naturally occurring signaling moieties, growth factors, and cytokines [49]. If on the one hand the use of a nanonized drug administered via the nose-to-brain route may facilitate its transport to the CNS overcoming the BBB; on the other hand the behavior of drug particles in the physiological brain environment has to be elucidated once delivered in the complex target site [5]. Moreover, we cannot exclude that part of the formulation may undertake the indirect route after intranasal administration and reach the brain by the systemic pathway. In order to evaluate the potential behavior of RSV_NC and pArgRSV_NC once delivered to the brain, preliminary particles’ colloidal stability was evaluated in a physiological environment. Specifically, variation in the RSV_NC and pArgRSV_NC mean size and PDI was evaluated in aCSF after incubation at 37 °C for 30 min, 1 h, and 24 h.

**Figure 7:**
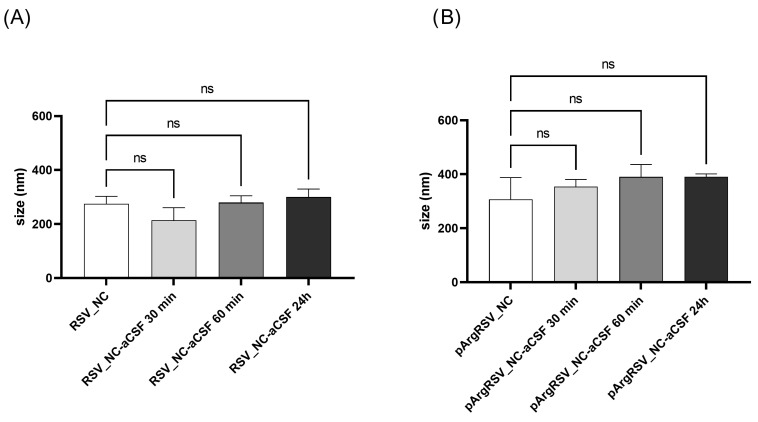
RSV NC (**A**) and pArgRSV NC (**B**) mean size after incubation in aCSF at 37 °C for 30 min, 1 h, and 24 h. *p* > 0.05 (not significant; ns).

No significant variations were observed for both formulations up to 24 h (Figure 7) after incubation in aCSF. Results suggested that no destabilization phenomena occurred despite the presence of ions in the artificial medium. PDI data partially support these findings since values were almost unchanged for RSV_NC < 0.375 and suffered an increase for pArgRSV_NC after 24 h (PDI = 0.7), suggesting the onset of slight aggregation. Colloidal stability under physiologically relevant conditions is critical for particle diffusion within the brain microenvironment and for the effective delivery of therapeutic agents to specific brain targets [50]. Overall, based on these results, both samples can be considered stable in aCSF, even if more specific tests are necessary to confirm their behavior in the biological milieu.

#### 3.3.5. Radical Scavenging Activity

**Figure 8:**
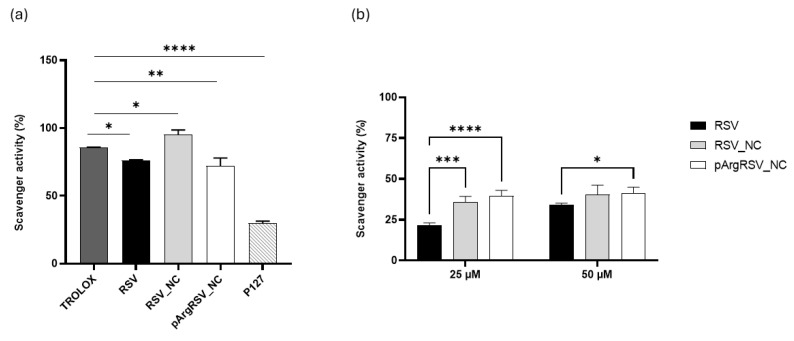
Radical scavenging activity % of (**a**) RSV, RSV_NC and pArg_RSV-NC. Results are expressed as mean ± SD (*n* = 3). **** *p* < 0.0001 vs. TROLOX (used as standard); ** *p* < 0.01 vs. TROLOX; and * *p* < 0.1 vs. TROLOX. (**b**) RSV, RSV-NC, and pArg_RSV-NC at 25 μM and 50 μM. Results are expressed as mean ± SD (*n* = 3). **** *p* < 0.0001 vs. RSV; *** *p* < 0.001 vs. RSV; and * *p* < 0.1 vs. RSV.

To evaluate the potential radical scavenging activity of RSV in the nanosized forms, a DPPH assay was conducted. The antioxidant activity of RSV was not negatively impacted by the nanonization process. RSV exhibited a scavenging activity of approximately 76%, which increased to around 95% inhibition with RSV_NC. Additionally, the formulation with pArg preserved this activity, indicating that neither the preparation process nor the cationic pArg functionalization affected the antioxidant potential (Figure 8a). The results demonstrated that the RSV formulated as NCs showed a greater radical scavenging capacity than raw RSV. A similar trend was reported by Jubori et al., specifically with RSV polymeric micelles [51]. It is well-known that RSV as a stilbene possesses significant scavenging activity. However, the addition of the surfactant P127 further enhanced the antioxidant activity compared to raw RSV. Poloxamer contains approximately 22 μg/g of Butylated hydroxytoluene (BHT), a recognized antioxidant [52], and its presence on the NC, resulting from the stabilizer coating, likely contributed to the increase in antioxidant activity beyond that of RSV alone (Figure 8a) [53]. Prior to biological assessment, the radical scavenging activity of both nanosuspensions was further investigated by the DPPH assay at the concentrations that will be tested on microglial cells. A similar trend was observed for RSV_NC and pArgRSV_NC at 25 μM and 50 μM (Figure 8b). RSV demonstrated a dose-dependent radical scavenging effect. The percentage of scavenging activity was maintained for both RSV_NC and pArgRSV_NC at these concentrations, highlighting their superior antioxidant activity compared to RSV alone. These preliminary results should be further confirmed in in vitro models of oxidative stress.

### 3.4. Cell Viability Analysis

Multiple studies have highlighted the importance of assessing the effects of natural molecules and peptides on various cell types under normal, non-stressful conditions. Compounds like RSV, oxyresveratrolcurcumin, piceatannol, tea polyphenols, and carnosine have been extensively investigated [54,55,56,57,58,59].

In the context of investigating formulations intended for intranasal administration, we commonly evaluate its cytotoxicity on olfactory ensheathing cells (OECs). These specialized glial cells envelop the axons projecting to the olfactory bulb and are thought to play a pivotal role in the direct transport of molecules to the brain following intranasal delivery. We already hypothesized that OECs display notable phagocytic capabilities and might exchange the engulfed content (i.e., nanosystems) with olfactory neurons.

Our previous studies demonstrated that NCs of carbamazepine and curcumin exhibit cytocompatibility with OECs and show enhanced cellular uptake compared to the free drug [4,5]. This improved uptake is likely attributable to the modified physico-chemical characteristics imparted by nanonization, particularly in terms of particle size, morphology, and surface hydrophilicity. Building upon these findings, the present study investigates the cytocompatibility of resveratrol nanosuspensions using a different cell line.

**Figure 9:**
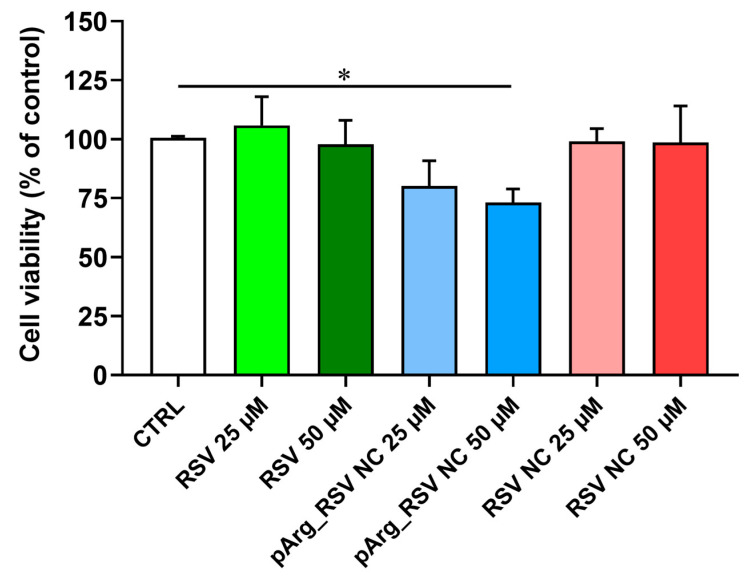
Cell viability analysis assessed by MTT assay. HMC3 cells were left untreated (CTRL) or treated with two different concentrations (25 and 50 µM) of RSV, pArgRSV_NC, or RSV_NC for 24 h. Data are the mean of 2 independent experiments (*n* = 8) and are expressed as the percent variation with respect to the cell viability recorded in CTRL cells. Standard deviations are represented by vertical bars. Significantly different, * *p* < 0.05.

Microglial cells are increasingly recognized as valuable therapeutic targets for several diseases, owing to their innate phagocytic function as immune cells [60]. Given their role in protein trafficking, aggregation, and clearance and their close involvement in neuroinflammation, microglia represent a promising focus for therapeutic intervention [60]. With this in mind, the last aim of the present study was to investigate whether RSV or its formulations (pArgRSV_NC and RSV_NC) were able to exert negative effects on HMC3 cell viability. Figure 9 shows the effects of RSV, pArgRSV_NC, and RSV_NC, all tested at two different concentrations (25 and 50 µM), on the viability of HMC3 cells. Literature findings revealed that RSV is highly effective in maintaining cell viability at concentrations ranging between 1 and 50 µM [61]. 

Neither RSV nor RSV_NC at both concentrations induced significant changes in cell viability compared to untreated (CTRL) cells. A different effect was observed only in the case of the higher concentration (50 µM) of pArgRSV_NC tested, which lead to a cell viability % value that was significantly different to CTRL cells (*p* < 0.05). This result could be due to the pArg effect, being a cell-penetrating peptide it facilitates RSV uptake and increases its intracellular concentration. These findings could be of interest not only for the present study, allowing the selection of the highest concentrations of RSV (50 µM), pArgRSV_NC (25 µM), or RSV_NC (50 µM) usable without toxic effects, but also for future studies considering microglia (HMC3), wherein the therapeutic potential of these compounds will be evaluated. Both RSV concentrations will be tested according to Han et al., who studied the neuroprotective effect of RSV against β-amyloid-induced neurotoxicity in rat hippocampal neurons and demonstrated that RSV (15–40 µM) significantly reduced Aβ25–35-induced cell death in a dose-dependent manner, with a maximal effect (93%) obtained at 25 µM [61].

## 4. Conclusions

RSV NCs were successfully designed and optimized using an I-Optimal design to construct a robust and accurate predictive model, thereby enhancing the scientific rigor and validity of our findings.

The sonoprecipitation approach affected the naked RSV NC morphology, appearing as truncated cubic crystals with a mean size of ~200 nm and a drug content of ~80%. RSV NC functionalization with the adsorption of the pArg homopolymer was efficiently performed, obtaining a positively charged nanosuspension. A saturated solubility assay demonstrated the ability of nanonization to increase drug solubility in water ~12-fold compared to the unprocessed RSV. Both nanosuspensions were stable at 5 °C up to 49 days in terms of particle size, PDI, and ZP. DSC and FTIR confirmed the absence of interactions between the components of each formulation and the effective coating of RSV NCs with pArg. Both nanosuspensions exerted mucoadhesive properties that were more pronounced for pArg RSV_NC, likely due to the combination of different mechanisms of action with pArg being a polycationic homopolymer acting as a mucoadhesive promoter, while P127 acts as a mucus-penetrating agent. The preliminary evaluation of the nanosuspension behavior in aCFS revealed their stability up to 24 h. The nanonization process did not negatively affect RSV radical scavenging activity, which was further enhanced. Furthermore, cell viability studies demonstrated the safety of all treatments in human microglia, except the highest pArgRSV NC concentration, setting the stage for future in vitro studies in which the neuroprotective activity of RSV and its formulations will be evaluated in microglial cells challenged with pro-inflammatory/pro-oxidant stimuli. Moreover, further investigations will be carried out to assess the in vitro permeability across nasal mucosa and the bioavailability of nanosized RSV following intranasal administration.

## Figures and Tables

**Table 1:** Factors and the corresponding levels investigated by the I-Optimal design.

Independent Variables	Type	Coded Factors	Polynomial Term	Levels	
				Low	High
Stabilizer conc. (%*w*/*v*)	numeric	X_1_	A	0.5	2
S/AS ratio (*v*/*v*)	numeric	X_2_	B	1:1	1:5
Stabilizer type	categoric	X_3_	C	Pluronic^®^ F127 Tween^®^ 80	
	**Dependent variables**				
Mean size (nm)		Y_1_			
PDI		Y_2_			
ZP (mV)		Y_3_			

**Table 2:** Composition and physico-chemical characteristics of optimized RSV NC.

RSV NC Composition			Optimized RSV NC		
	Stabilizer	S/AS Ratio (*v*/*v*)	Mean Size (nm ± SD)	PDI ± SD	ZP (mV ± SD)
Conc. (% *w*/*v*)	Type				
0.7	P127	1:2	245.2 ± 27.6	0.30 ± 0.12	−23.7 ± 0.4

## Data Availability

Dataset available on request from the authors.

## Supplementary Materials

The following supporting information can be downloaded at https://www.mdpi.com/article/10.3390/pharmaceutics17101346/s1. Figure S1: (a) SEM micrograph showing the overall morphology of the NC; (b) SEM detail showing the morphology of a single particle; (c) Diameter distribution histogram (green bars) obtained by averaging different SEM images, fitted with a Gaussian function (red line); Table S1: Combination of independent variables in RSV NC experimental runs prepared according to I-Optimal design; Table S2: RSV NC optimization.
