# Supplementary material for: Design of Polycation-Functionalized Resveratrol Nanocrystals for Intranasal Administration
Source: Pharmaceutics. 2025 Oct 18;17(10):1346. doi: 10.3390/pharmaceutics17101346 (PMC12566871; doi:10.3390/pharmaceutics17101346)
Supplement: Supplementary file 1 [file pharmaceutics-17-01346-s001.zip › pharmaceutics-3905619-supplementary.pdf]

pharmaceutics

Supplementary Materials: Design of Polycation-Functionalized Resveratrol Nanocrystals for Intranasal Administration

Angela Bonaccorso, Elide Zingale, Giuseppe Caruso, Anna Privitera, Claudia Carbone, Maria Josè Lo Faro, Filippo Caraci, Teresa Musumeci and Rosario Pignatello

Table S1. Combination of independent variables in RSV NC experimental runs prepared according to I-Optimal design.

Run	Stabilizer conc. (%w/v)	S/AS ratio (v/v)	Stabilizer type
1	1	1:2	Tween 80
2	1	1:2	Tween 80
3	2	1:1	Tween 80
4	2	1:5	Pluronic F127
5	1	1:2	Tween 80
6	1	1:2	Tween 80
7	0.5	1:5	Pluronic F127
8	0.5	1:2	Pluronic F127
9	1	1:5	Pluronic F127
10	2	1:2	Tween 80
11	1	1:1	Pluronic F127
12	1	1:1	Tween 80
13	0.5	1:1	Tween 80
14	2	1:2	Pluronic F127
15	1	1:2	Pluronic F127
16	2	1:5	Tween 80
17	0.5	1:5	Tween 80

Table S2. RSV NC optimization.

Factors and Response	Goal	Lower limit	Upper limit
Stabilizer conc. (% w/v)	maximize	0.5	2
S/AS ratio (v/v)	in the range	1:1	1:5
Type of stabilizer	equal to	Pluronic®F-127	
Mean size (nm)	minimize	200	500
PDI	minimize	0.261	1.00
ZP (mV)	minimize	-17.5	+1.97

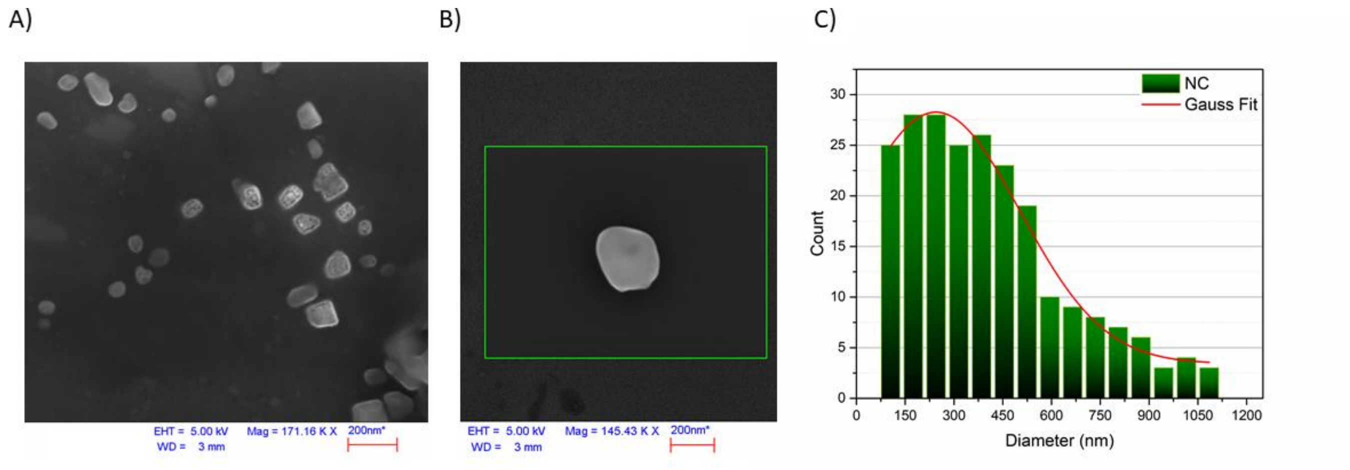

Figure S1. (a) SEM micrograph showing the overall morphology of the NC; (b) SEM detail showing the morphology of a single particle; (c) Diameter distribution histogram (green bars) obtained by av-eraging different SEM images, fitted with a Gaussian function (red line).
